# On Structural Rearrangements Near the Glass Transition Temperature in Amorphous Silica

**DOI:** 10.3390/ma14185235

**Published:** 2021-09-11

**Authors:** Michael I. Ojovan, Robert F. Tournier

**Affiliations:** 1 Department of Materials, Imperial College London, South Kensington Campus, Exhibition Road, London SW7 2AZ, UK; 2 Department of Radiochemistry, Moscow State University Named after M.V. Lomonosov, Leninskie Gory 1, Bd.3, 119991 Moscow, Russia; 3 Laboratoire National des Champs Magnétiques Intenses, European Magnetic Field Laboratory, UPR 3228 Centre National de la Recherche Scientifique, Université Grenoble Alpes, Institut National des Sciences Appliquées de Toulouse, F-31400 Toulouse, France; robert.tournier@lncmi.cnrs.fr

**Keywords:** amorphous silica, structure, glass transition, chemical bond, percolation, Hausdorff-Besicovitch dimension

## Abstract

The formation of clusters was analyzed in a topologically disordered network of bonds of amorphous silica (SiO_2_) based on the Angell model of broken bonds termed configurons. It was shown that a fractal-dimensional configuron phase was formed in the amorphous silica above the glass transition temperature T_g_. The glass transition was described in terms of the concepts of configuron percolation theory (CPT) using the Kantor-Webman theorem, which states that the rigidity threshold of an elastic percolating network is identical to the percolation threshold. The account of configuron phase formation above T_g_ showed that (i) the glass transition was similar in nature to the second-order phase transformations within the Ehrenfest classification and that (ii) although being reversible, it occurred differently when heating through the glass–liquid transition to that when cooling down in the liquid phase via vitrification. In contrast to typical second-order transformations, such as the formation of ferromagnetic or superconducting phases when the more ordered phase is located below the transition threshold, the configuron phase was located above it.

## 1. Introduction

Silica is the most common oxide on the Earth, characterized by a mean abundance of about 37 wt%. Amorphous silica exists either as glass while the temperature is below the glass transition temperature, which is T_g_ = 1480 K at normal pressure, or as a melt above this temperature. Amorphous silica has a three-dimensional interconnected structure of SiO_4_ tetrahedra up to about 15 GPa, with an average Si–O–Si bond angle of 144–153°, which decreases by about 1° per 100 K with an increase of temperature above T_g_ at the ambient pressure [1]. The silica melt is thermodynamically stable above the melting temperature, which is T_m_ = 1986 K, while below this temperature, both melt and glass are metastable structures. The changes in the structure that occur at T_g_ when a metastable liquid vitrifies into a glass are of general interest, and investigations in this area persist, including works that are related to understanding the role of a modified random network in glasses and the glass transition, where silica is a relatively simple system for analysis [2,3,4,5,6].

Glass is an amorphous substance with the mechanical properties of an isotropic solid; it is found in nature and is used in various fields of human activity. However, both the nature of the glassy state and glass transition at the atomic-molecular level are not well understood [7,8,9,10,11,12,13,14,15]. At present, there is no unified approach to the physical nature of the glass transition. Moreover, the glass transition is often not considered as a thermodynamic phase transition; instead, a rule of thumb is introduced that states that an amorphous material is considered to be glass if its viscosity is equal to or higher than 10^12^ Pa·s (10^13^ poise) [16], although the glass transition temperature T_g_ is in practice determined from the characteristic kink of the temperature dependences of the specific volume or enthalpy at T_g_ [7]. Although the glass transition manifests itself as a typical second-order phase transformation and the derivative thermodynamic parameters of amorphous materials, such as the thermal expansion coefficient or the heat capacity, experience a characteristic jump exactly at T_g_, its description in terms of Landau’s theory is not an easy task, e.g., there is no clarity about the order parameter describing this transition.

Significant efforts are continuing to reveal the thermodynamic origins of the glass transition in various materials, including oxides, metals, organics (see, e.g., [17,18,19,20,21,22,23,24,25]), and the newly discovered Metal–organic framework (MOF) glasses [26]. Molecular dynamics (MD) experiments show that below T_g_, percolation clusters are formed in amorphous materials made up of Voronoi coordination polyhedra of high-density atomic configurations [27,28]. Both the analytical approach and MD simulations also show that, during the glass transition, the geometry of an amorphous material changes due to the formation of macroscopic percolation clusters [27,28,29], and percolation clusters are formed in the liquid state made up of Voronoi coordination polyhedra of low-density atomic configurations, while in the solid (glassy) state, there are no such clusters [27]. In many works, structural analysis was shown to be a powerful tool for understanding the changes that occur in an amorphous material at the glass transition [30,31], e.g., analysis of X-ray diffraction (XRD) patterns of amorphous materials shows a step-wise change of the slope of the temperature dependence of the first sharp diffraction minimum exactly at T_g_ [25]. We note that similar methods can be used to study the structural rearrangement of other amorphous materials at the glass transition. 

Glasses are isotropic and homogeneous at the macroscopic scale; however, the structure of glasses comprises the following attributes: (i) short-range order (SRO) with molecular-type units, i.e., building blocks, such as tetrahedral structures in silicates at the atomic scale; (ii) medium-range order (MRO) at a larger size range, which extends from second- and third-neighbor environments to percolating and fractal structures; and (iii) a disordered state (DS), which is homogeneous and isotropic, as observed at macroscopic sizes [22,32,33]. A diverse variety of glasses exist with a wide range of structural SRO building blocks from which the DS is composed of. The connections between the building blocks of glasses occur via the chemical bonding system. Typical oxide glasses have SRO building blocks that are connected via covalent and heteropolar (mixed covalent-ionic) bonding, whereas chalcogenide glasses are built using covalently bonded two-, three-, and four-coordinated SRO building blocks. Metallic glasses consist mainly of SRO icosahedral building blocks that are interconnected via metallic bonding [28]. Organic glasses have cross-linked polymeric molecular chains as SRO building blocks and van der Waals bonds between them. Glassy water has tetrahedral units as SRO building blocks, which are connected via hydrogen bonds.

The silica glass in normal conditions forms a fully polymerized network structure of corner-sharing tetrahedral SiO_4_ units and its structure is characterized by a random distribution of chemically ordered rings, where the silicon atoms are linked by bridging oxygen atoms [32]. As such, the amorphous SiO_2_ looks like a relatively simple system with covalent bonds connecting the silicon with oxygens and thus allowing for more easily revealing the changes that occur due to the increase in temperature above T_g_ when a glass transforms into a liquid.

A liquid phase can be characterized as a dynamically homogeneous medium since there is always a tendency in it to form microscopic metastable ordered structures. Moreover, with a decrease in temperature, the sizes and lifetimes of these structures increase. On large time and space scales, the liquid is homogeneous and disordered, but locally, at the size of metastable clusters and during the lifetime of metastable formations, the liquid can have an SRO as well. Local ordering in supercooled liquids is confirmed by Fisher clusters, which are associated with long-wave density fluctuations and are revealed in glass-forming liquids and polymers [34]. With correlation lengths up to 300 nm, density fluctuations turned out to have a fractal structure with a dimensionality less than 3 [35]. Experimental observations confirmed the presence of local ordering in liquids, e.g., ordered structures were found above the liquidus of silicate melts [36]. Furthermore, direct observation of the spatial distribution of microparticles of emulsions confirmed the dynamic heterogeneities of liquids [37]. Thus, the structures of disordered materials in the liquid and solid states are significantly different. Although macroscopically, the distribution of atoms or molecules is topologically disordered both in the glassy and in the molten state, in the low-temperature (non-ergodic) region, the structure of the amorphous material is rather solid-like and the geometric structure of the interatomic (intermolecular) bonds is three-dimensional with a small fraction of defects in the form of broken chemical bonds termed configurons. Above T_g_, i.e., in the ergodic region, the structure is characterized by the presence of fractal dynamic molecular structures up to and even above the liquidus temperature, as well as macroscopic clusters that are made up of configurons.

The aim of this work was to analyze the structural changes at the glass–liquid transition. We focused here on amorphous SiO_2_ since we could trace the topological characteristics of the covalent bonding system. We showed that the glass transition led to the formation of a configuron phase above T_g_ that had a fractal structure and grew with the increase in temperature. The glass transition described in terms of the configuron phase formation above T_g_ corresponded to a second-order phase transformation within the Ehrenfest classification.

## 2. Configurons

**Figure 1:**
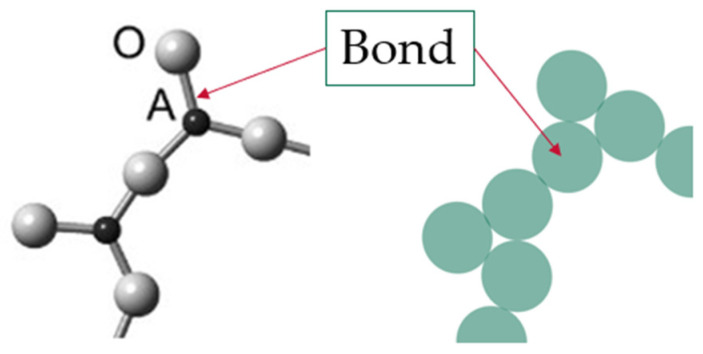
Example of a congruent bond lattice (CBL) model for an A_2_O_3_ glass. The structure of glass composed of A^2+^ cations connected via bridging O^2−^ anions is replaced by the CBL shown in green color, with bonds that are assumed to take a spherical form.

The term configuron was coined by Angell and co-authors, who introduced the congruent bond lattice (CBL) model with the aim of replacing the set of atoms with a congruent structure of weakly interacting bonds that is easier to analyze [38,39,40,41]. The CBL model is applicable to the case of any isotropic material, including crystalline substances, to understand the related transition because any condensed phase can be represented by a three-dimensional array of the interactions between its particles [41]. In the CBL, the system of weakly interacting chemical bonds congruently represents the initial system of strongly interacting ions, which are Si^4+^ and O^2−^ ions in the amorphous SiO_2_. Figure 1 illustrates the introduction of CBL for the glassy A_2_O_3_ system. 

**Figure 2:**
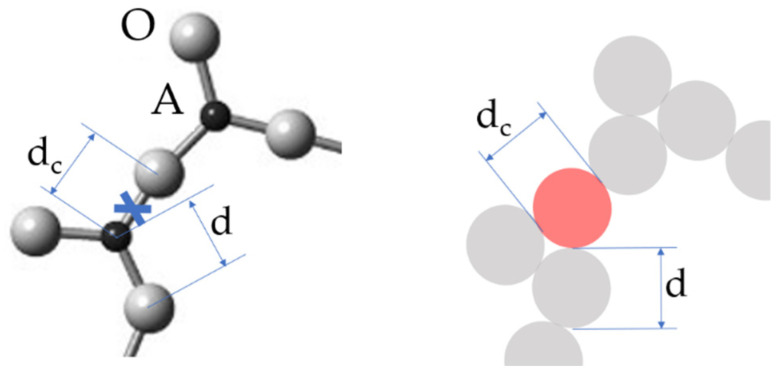
Schematic of a configuron (shown in light red) in the A_2_O_3_ glass. The configuron diameter d_c_ is not necessarily equal to the initial bond length d.

At finite temperatures T > 0 some of the chemical bonds are broken; moreover, the higher the T, the more bonds are broken. Each broken bond, along with the associated strain-releasing local adjustment of centers of atomic vibrations, is treated following Angell as an elementary excitation termed a configuron. Figure 2 illustrates the formation of a configuron in an A_2_O_3_ glass.

The sizes d_c_ of newly formed configurons, which are assumed to be spherical, are not necessarily equal to the initial bond lengths d, which is 1.62 Å in silica. They are typically slightly larger d_c_ > d; however, they can be of the same size as the initial bond, as well as slightly smaller d_c_ < d. It is important that the notion of a configuron is not limited to only well-localized bonds, such as those of ionic or covalent type, and can be used for metallically bonded materials, i.e., for metallic glasses as well (Table 1).

**Figure 3:**
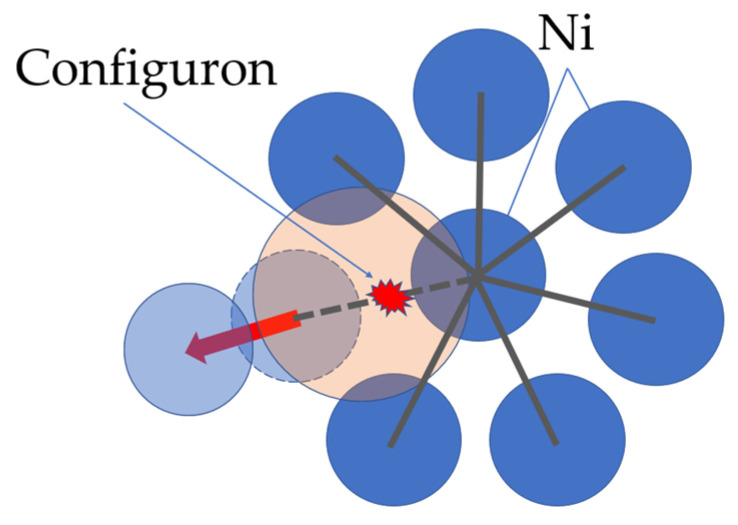
Configuron formation in amorphous nickel due to a change in the local atomic connectivity by losing one nearest neighbor in the first coordination shell. Reprinted with permission from [25]. Copyright 2020 American Chemical Society.

Indeed, when atomic vibrational motions become anharmonic enough, the probability is high that groups of particles in an amorphous material will have simultaneously moved far enough from their original positions that it becomes energetically unfavorable for them to return, which corresponds to crossing of a local potential barrier. This motion affecting neighboring particles as concomitant shifts, which occur in the centers of vibration of other particles in the vicinity such that adjustments to the primary motion spread out through the material. As the new configuration will generally differ in energy from the initial one, the difference corresponds to a configuron that is an excited configurational state for the system. Phonon momentum but not energy will be conserved in such processes [38]. In the case of metallic glasses, the configuron as an elementary excitation is based on Egami’s ideas of changes of the local atomic connectivity by losing or gaining one nearest neighbor [42], see Figure 3. 

The configuron that is formed does not support particles that are bound to each other; therefore, instead of an elastic bond, the rigid connection between particles disappears and the previously bound particles can move away. With an increase in temperature, more and more bonds are broken until percolation via broken bonds occurs. As shown by Kantor and Webman [43], the rigidity threshold of an elastic percolating network is identical to the percolation threshold. Thus, the formation of the percolation cluster made of configurons results in a kind of disintegration loss of rigidity or, in other words, to a transformation of a solid to a liquid; hence, the glass transition occurs and the glass, which is a rigid elastic solid, transforms to a plastic liquid. The transformation of a glass into a liquid, i.e., the glass transition, is described based on the CBL model, which was specifically designed to treat the glass transition phenomena. This is done using the general methodology of percolation theory, i.e., the concept of configuron percolation theory (CPT).

## 3. Glass Transition in Silica

To consider the amorphous SiO_2_ structure, we used the CBL where instead of the vitrification of molten material, we started with its solid state, i.e., the glass, and considered its melting, tracing the changes in the spatial distribution of configurons due to a gradual increase in temperature from zero to temperatures above T_g_. The bond system forms a topologically disordered lattice that is congruent to the disordered lattice of the amorphous SiO_2_. Assuming only two allowed states for bonds, namely, binding (ground) and broken (excited), allowed us to take advantage of the statistics of two-level systems and simplified further formalism [44,45]. The system of N strongly interacting ions was replaced by a system of N’ = NZ weakly interacting bonds, where broken bonds were viewed as antibonds with energy equal and opposite to those of bonds, where Z is the coordination number (Z = 4 for SiO_2_). Note that for amorphous materials in which cations are not bonded via bridging atoms, such as bridging oxygens in silica or chlorine in ZnCl_2_, e.g., for amorphous Fe, the number N’ = NZ/2. The distance between the cation and the oxygen anion in the SiO_4_ coordination tetrahedron of amorphous SiO_2_ is d = 1.62 Å; thus, the volume V_0_ = π d^3^/6 could be assigned to each bond. The configuron volume V is typically larger than V_0_; thus, the change in molar volume of material V_m_(T) at finite temperatures is typically positive and is proportional to the number of configurons formed:

V_m_(T) = V_mv_(T) + V_c_(T) (1)

where V_mv_(T) is the molar volume of the material when considering only vibrations [46,47] and V_c_(T) is the molar volume of configurons, which is given by:

V_c_(T) = N_c_(T)ΔV (2)

where, N_c_(T) is the number of configurons that are formed at temperature T, and the added volume due to the configuron formation is:

ΔV_0_ = V − V_0_ (3)

Typically, the added volume is positive ΔV_0_ > 0; however, this is not an a priori condition and the materials that have the volumes of configurons V = π d_c_^3^/6 smaller than the initial bond volumes, i.e., ΔV_0_ < 0, will exhibit a negative temperature expansion coefficient when the negative added volume is considerable. 

The fraction of configurons that are formed or the degree of breakage of the material network as a function of temperature is determined according to statistical thermodynamics using the expression [29,44,45]:

f(T) = exp(−G_c_/RT)/[1 + exp(−G_c_/RT)] (4)

where G_c_ = H_c_ − TS_c_ is the free Gibbs energy of the configurons, H_c_ is the enthalpy of formation, S_c_ is the entropy of formation of the configurons, and R is the universal gas constant. The number of configurons is thus:

N_c_(T) = ZNf(T) (5)

Configurons that are generated, annihilated, and moving at relatively higher concentrations associate with each other, which is a generic feature of any collection of independent particles that move, reproduce, and die. Indeed, such collections may undergo wild fluctuations at the local and global scales, inducing characteristic patchiness in the spatial distribution of the individuals observed in the spread of epidemics [48,49,50], the growth of bacteria on Petri dishes [51,52], the dynamics of ecological communities [53], the mutation propagation of genes [54], susceptible–infected–removed spreading processes [55], and in the distribution of neutrons in nuclear reactors, which was recently shown to be an effect of clustering [56]. As long as there is a tiny number of small clusters made up of configurons, they can be neglected; however, as the temperature increases, there are more configurons and configuron clusters, which grow in size, and when the threshold value determined by percolation theory [29,57] is reached, a macroscopic percolation cluster of configurons is formed in the system, which penetrates the entire volume of the material. The macroscopic cluster of configurons changes the behavior of the bond system since a path of facilitated motion of atoms appears. Based on Frenkel’s [58] and Trachenko et al.’s [59,60,61,62] works, we conclude that when additional availability for atomic motions is ensured, the material shifts from solid-like to gas-like type behavior. In line with Benigni’s statement on liquid-like (the B phase in the two-state model) [63], the state of atoms, which are included in the percolation cluster made up of configurons, is assimilated into a gas-like type with consequent contributions to the heat capacity of material and its mechanical properties. Thus, the glass–liquid transition temperature can be found from the condition of reaching the percolation threshold f_c_ [29,44,45]:

f(T_g_) = f_c_ (6)

For the glass transition temperature the equation, this gives:

T_g_ = H_c_/{S_c_ + R ln[(1 − f_c_)/f_c_]} (7)

The threshold f_c_ in the idealized case can be taken to be equal to the critical packing density for the three-dimensional space given by the Scher and Zallen invariant [57,64]:

ϑ_c_ = 0.15 ± 0.01 (8)

The thermodynamic parameters of configurons in amorphous silica are known: H_c_ = 237 kJ/mol and S_c_ = 17.54 R [45], which gives the glass transition temperature of amorphous silica: T_g_ = 1475 K. This temperature practically coincides with the experimentally known temperature T_g,exp_ = 1480 K [1], especially when accounting for the experimental errors [32,65]. The universal critical threshold f_c_ = ϑ_c_ works well for silica and germania [44,45], as well as for many metallic systems [66]; however, for complex oxide systems, f_c_ << 1, which indicates that the percolation threshold is reached at much lower concentrations of broken bonds and the effective configuron radii in these systems are larger [45,67] or there is patchiness of configuron clustering following the generic pattern of dynamic systems [48,49,50,51,52,53,54,55,56]. The formation of stable and ultrastable glasses leads to higher glass transition temperatures. Their liquid states are changed after heating them above T_g_. Consequently, the percolation threshold is increased. Glacial phases have still higher transition temperatures T_g_ with higher percolation thresholds. These events occur for singular values of enthalpy [23,66], introducing more percolation thresholds. These threshold values must be singular to represent various organizations of elementary bricks. 

The percolation cluster does not exist below T_g_; it appears in the system when the temperature reaches T_g_, growing further with an increase in temperature. The density of the percolation cluster of configurons is also a universal property that is determined by the percolation theory using:

φ(T) = p[f(T) − f_c_]^β^ Θ(T − T_g_) (9)

where p is a numerical coefficient close to one, Θ(x) is the Heaviside step function, and β = 0.41 is the critical index in the three-dimensional space [57]. Note that close to T_g_, we have approximately (f(T) − f_c_) = (T − T_g_) f_c_ (1 − f_c_)) H_c_/RT_g_^2^ [44]. The percolation cluster conventionally penetrates the whole volume of material, dividing it into cells with a size that is the so-called correlation length:

ξ(T) = ξ_0_/|f(T) − f_c_|^ν^ (10)

where ν = 0.88 is the critical index in the three-dimensional space [57]. The ξ_0_ is an elementary length that is approximately equal to the bond length d. As the percolation threshold is approached, we have f(T) → f_c_, and the correlation length diverges. This reflects the fact that the percolation cluster of broken bonds penetrates the entire volume of an amorphous material. At sizes that are significantly larger than ξ(T), the material can be considered as homogeneous, while at sizes that are similar to or smaller than ξ(T), the material has an inhomogeneous structure, which is described using fractal geometry. As the correlation length equalizes, the sample size L at the glass transition temperature is lower compared with T_g_ of large size samples and is given by [44]:

T_g_(L)=T_g_ − 0.1275 T_g_ (RT_g_/H_c_)(ξ_0_/L)^1.136^ (11)

This result is in line with the known size dependence of the glass transition temperature (T_g_ − T_g_(L)) ∝ 1/L [68] and enables an explanation of the size effects of glass transition in thin films [69]. It can also shed light on the effects that are observed in phase-separated glasses, where the changes in T_g_ may be partly due to the finite size effects of the glass transition [70,71,72,73,74]. 

**Figure 4:**
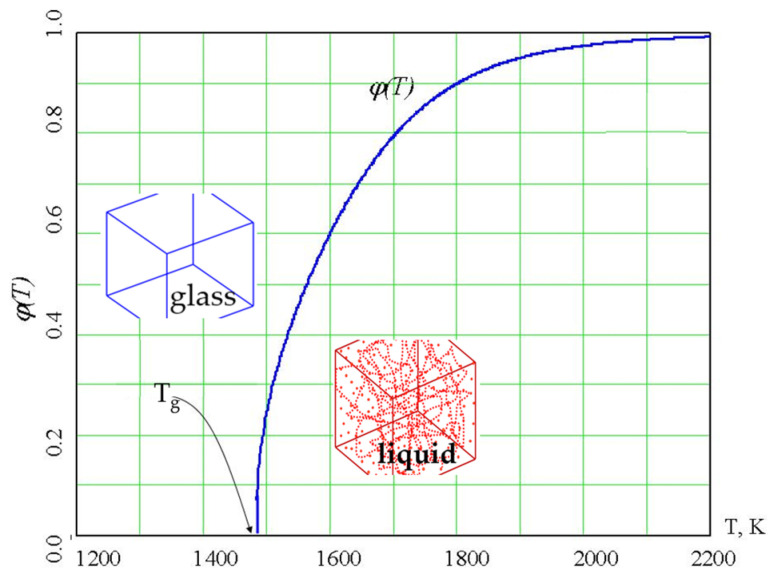
The order parameter of configurons φ(T) in amorphous SiO_2_. Configurons occur in the glass as point defects, whereas in the liquid phase (above the T_g_), they form macroscopic percolating clusters, as schematically shown by the insets.

A percolation cluster is a fractal object that has the Hausdorff dimension [57] D = 2.55 ± 0.05. The density of a percolation cluster made of configurons (Equation (9)) changes from 0 to 1, with it being equal to 0 up to T_g_ and then growing until it reaches 1 (Figure 4), and was proposed to be considered as an order parameter in the sense of the general Landau theory of phase transformations [44,75]. 

The number of configurons belonging to the percolation cluster formed above T_g_ is calculated according to percolation theory:

N_p_(T ≤ T_g_) = pZN[f(T) − f_c_]^β^ (12)

The volume of a configuron in the percolation cluster V’ is not the same as the volume of a separated configuron V; therefore, (V’ − V) ≠ 0 and there is an additional change of volume ΔV due to configurons that belong to the percolation cluster:

ΔV = (V’ − V)(13)

This change is not necessarily positive for any material, although it is typically large ΔV > 0, e.g., the increase in volume of ZnCl_2_ is ΔV = 6.9 mL/mol [40]. 

Thus, above T_g_, the equation for the temperature dependence of the volume change of materials (Equation (1)) needs to be modified to account for the percolation cluster formed in the liquid phase:

V_m_(T) = V_mv_(T) + V_c_(T) + V_pc_(T) (14)

**Figure 5:**
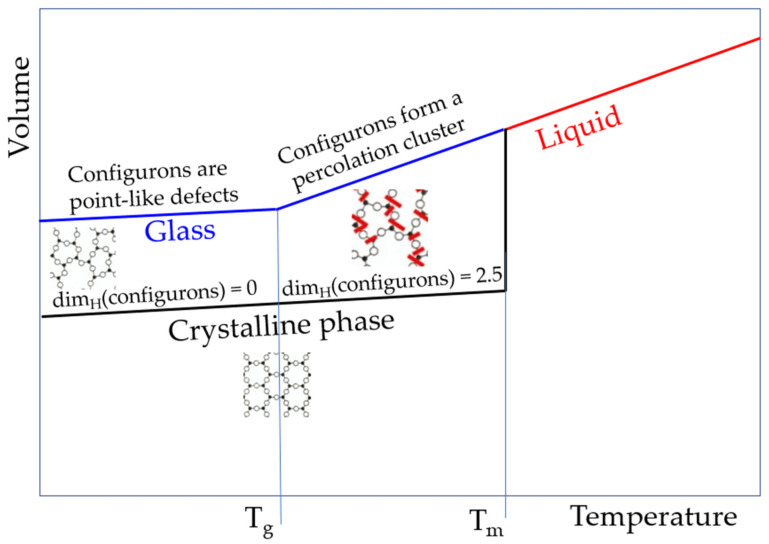
Schematic of specific volumes of stable glass (T < T_g_), supercooled melt (T_g_ < T < T_m_), and stable melt (T > T_m_). For silica, T_g_ = 1980 K and T_m_ = 1986 K. The molar volume of silica melt is 27.4 cm^3^/mol. Note that the slope of lines is significantly exaggerated for the amorphous silica to account for the very low expansion coefficient, which is not higher than 4.9 × 10^−6^ K^−1^ for the glassy phase and not higher than 7 × 10^−6^ K^−1^ for the melt [1].

The volume added by the percolation cluster is:

V_pc_(T) = ΔV pZN[f(T) − f_c_]^β^ (15)

which accounts for the volume of a configuron in the percolation cluster not being the same as the volume of a separate configuron. Hence, the dependence of the molar volume on temperature, including the configurational contributions (Equation (12)) does not experience an abrupt change and remains a continuous function of temperature, although it exhibits a kink at T_g_ due to contributions of the term in Equation (13) that results from the formation of the percolation cluster made of configurons. Figure 5 depicts a schematic of the volume change of amorphous and crystalline materials due to an increase in temperature. Note that the antibond formation in the excited state does not modify this description of the volume change above T_g_.

## 4. Discussion

The configurational entropy of a material is determined by the number of configurons Nc that are accessible to it at the given temperature T, as follows [76]:

S_c_ = k_B_ lnW = k_B_ ln[N_c_(T)] (16)

where k_B_ is the Boltzmann constant, W is the total number of distinct packing states that are available to a system, and W = N_c_(T) while T < T_g_. This term exists at any finite temperature T > 0 and cannot be arbitrarily set to zero as is most often done in the two-level models, such as that described in [63]. However, above T_g_, a new term needs to be added to the configurational entropy of liquid (Equation (16)), which is due to the formation of the percolation cluster that is made up of configurons, where atoms exploit their new degree of freedoms as more space becomes available for the cluster’s motion:

S_pc_ = k_B_ ln[N_p_(T)] (17)

**Figure 6:**
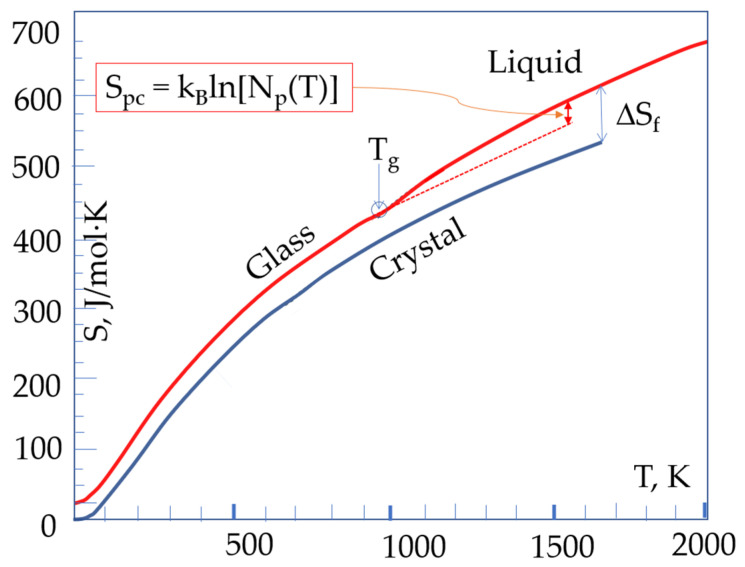
The temperature dependence of the entropy of amorphous and crystalline phases of diopside with the explicit contribution of a configuron percolation cluster above the T_g_. ΔS_f_ shows the entropy difference between the liquid and the crystalline phases at the melting temperature.

Figure 6 demonstrates the natural appearance above T_g_ of the contribution of configurons that are associated with the percolation cluster for amorphous diopside, where the numerical data is taken from [32] (p 257). The existence of two variations of the entropy above T_m_ is shown because two values of enthalpy exist up to the temperature T_n+_: 

(i) The effective entropy S is decreased at T_n+_ (θ_n+_ = (T_n+_ − T_m_)/T_m_):

ΔS (θ_n+_) = H_m_/T_m_ − θ_n+_ H_m_/T_n+_ = S_m_ (1 − θ_n+_/(1 + θ_n+_)) (18)

(ii) The effective entropy S is increased at T_n+_:

ΔS = H_m_/T_m_ + θ_n+_H_m_/T_n+_ = S_m_ (1 + θ_n+_/(1 + θ_n+_)) (19)

Consequently, the entropy variation at T_n+_ has two values

ΔS (θ_n+_) = ±S_m_θ_n+_/(1 + θ_n+_) (20)

V_pc_ (T) can be positive or negative. It is negative for systems that are annealed between T_m_ and T_n+_ or slowly cooled, favoring bond formation. It is positive for quenched systems, favoring antibonds when an enthalpy increase corresponds to a volume increase. The sign of this quantity depends on the heating and cooling rates and mainly on the thermal history above T_m_.

The account of configuron phase formation above T_g_ hence shows that the glass transition is similar to the second-order phase transformations within the Ehrenfest classification [77,78,79], which is based on the change of state variables at the transition temperature T_tr_. The second-order transitions are continuous for the volume, the enthalpy, and the entropy, which show a kink at the T_tr_, while the second derivative of the chemical potential and heat capacity change discontinuously. In line with the Landau theory of second-order phase transformations, they occur when a new state of reduced symmetry develops continuously from the more disordered phase, which is typically at the higher temperature. He observed that near a phase transition, an approximate form for the free energy can be constructed with an order parameter that is zero before the transition and nonzero after the phase transition [79]. Examples of second-order transitions include the ferromagnetic–paramagnetic transition, where the magnetization is the order parameter and the structural phase transition from a cubic phase to a tetragonal phase occurs, where the order parameter is the parameter (c/a − 1), where c is the length of the long side of the tetragonal unit cell and a is the length of the short side of the tetragonal unit cell. For second-order phase transitions, the order parameter increases continuously from zero, starting at the T_tr_. As we see from Equation (9) and Figure 2, the transition of a glass to a liquid at T_g_ exactly follows the universal case of second-order phase transformation, although it occurs in the metastable phases as both a glass and a supercooled liquid are not thermodynamically equilibrium states of matter. Due to the formation of the configuron phase, the specific heat capacity of amorphous materials at the transition follows the universal law:

C_pc_(T) = C_0_/|T − T_g_|^1−β^ (21)

where C_0_ is a coefficient that accounts for the contribution of the configuron phase. As (1 − β) = 0.59, the heat capacity contribution from the formation of configuron phase diverges at T_g_, which, in practice, is used to determine T_g_ of glass-forming liquids. The same temperature dependence is true for the thermal expansion coefficient of amorphous materials. This type of behavior of heat capacity and thermal expansion allows for an exact identification of T_g_ when increasing the temperature through the transition of glass into a liquid.

The configurons are quasiparticles that are specific to initially solid materials (glasses) and can only be properly identified when the system is not too far from the percolation threshold and therefore not significantly above the T_g_; the system loses any individuality at T_m_ and above it. The vitrification of liquids occurs when cooling down, which induces the formation of new bonds rather than configurons until the system finally achieves the rigidity threshold, which is the same as the percolation threshold following Kantor–Webman theorem [43]. The vitrification is not associated with discontinuities in the temperature dependence of heat capacity, i.e., when decreasing the temperature, because melt vitrification is not accompanied by configuron phase formation. The contribution from the annihilation of configurons, which is the same as bond formation when decreasing the melt temperature, has an opposite sign (exo- instead of endo-) to that of increasing the temperature. Hence, the CPT naturally explains the experimentally known hysteresis in the behavior of heat capacity near the glass transition when increasing or decreasing temperatures near T_g_. Notably, an account of the clusters formed in the liquids enables an explanation of liquid–liquid phase transformations and the Mpemba effect and its inverse, which exists in all melts. The complete annihilation of configurons occurs by heating above the temperature T_n+_ > T_m_ toward the homogeneous liquid state. Each configuron has the energy of an antibond, which is equal in size but with an opposite sign to that of a bond that would appear by annealing the homogeneous liquid between T_m_ and T_n+_ and would disappear by heating above T_n+_ [80].

Figure 4 reveals an atypical feature of the glass transition within CPT, namely, the location of the more ordered configuron phase above the T_tr_. The unusual location in the higher temperature range is due to the increase in configuron concentration with temperature. This makes the glass–liquid transition rather similar to the formation of condensed Rydberg matter at high levels of excitation of atoms [81,82], although the condensation of excited atoms into condensed Rydberg matter occurs via a first-order phase transition. Notably, the glass transition can be interpreted in terms of first-order transformations [12,17,20,23].

The configuron phase that is formed exactly at T_g_ grows with an increase in temperature above T_g_. Set theory, which is a branch of mathematical logic that studies abstract sets as collections of objects [83], can be used to characterize the configuron phase, where the Hausdorff-Besicovitch dimension is defined as the limit:

(22) $$\dim_{H}(\mathrm{set})=D=\lim_{\varepsilon \to 0}[\frac{\log N(\varepsilon )}{\log(1/\varepsilon )}]$$

where N(ε) is the number of boxes of side length ε that are required to cover the set of configurons. It is concluded that the set has the Hausdorff-Besicovitch dimension D when N(ε) grows proportionally to 1/ε^D^ as ε tends to zero. 

**Figure 7:**
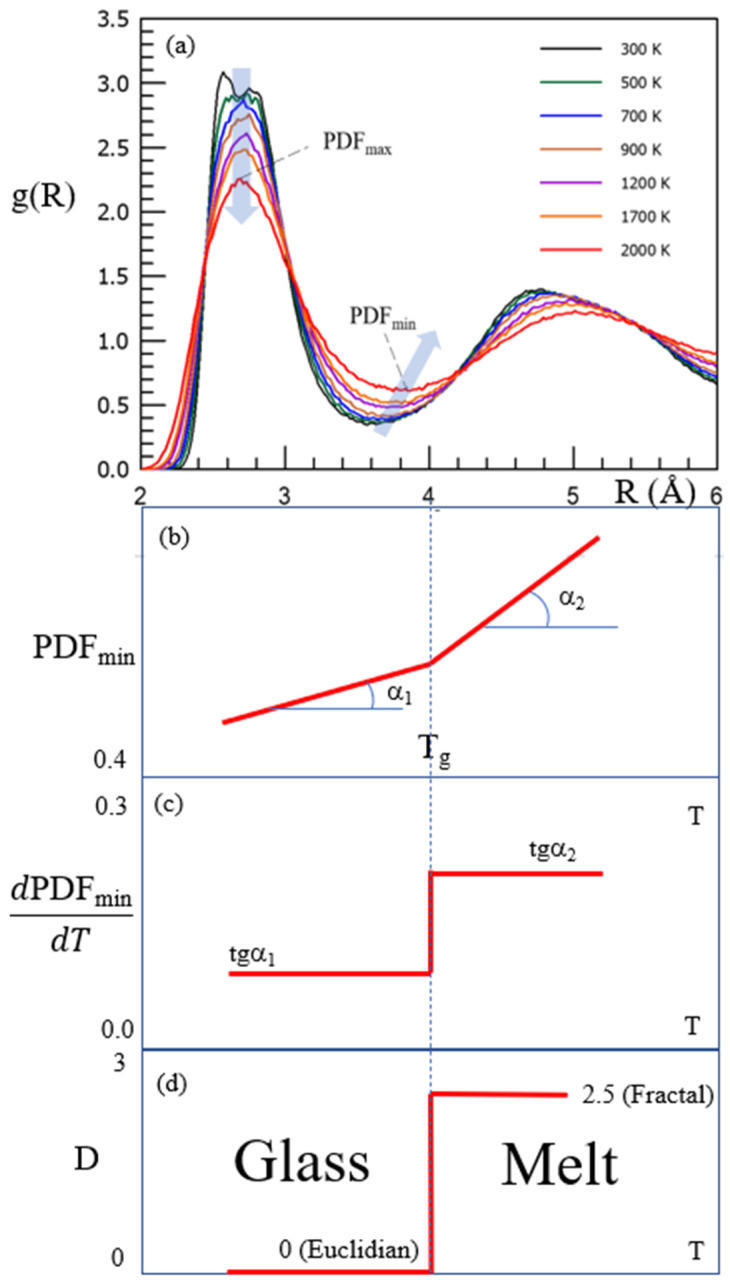
Schematic of the pair-distribution function PDF(R) = g(R) of amorphous Ti_2_Ni (**a**). Reprinted with permission from [25]. Copyright 2020 American Chemical Society. The prominent changes during the glass transition of PDF_min_ (**b**,**c**) and the Hausdorff-Besicovitch dimension of configurons set (**d**).

As shown above in Figure 5, the dimensionality of the set of configurons (configuron phase) changes at T_g_ from 0 to D = 2.55 ± 0.05 above it [67]. This stepwise change of dimensionality of the set of configurons is due to the formation of a configuron phase above T_g_ and has a kink in the first sharp diffraction minimum of scattered X-ray or neutrons as a consequence [15,25]. The presence of this kink gives evidence of structural differences between glasses and melts and is used in practice to identify T_g_ of metallic systems [25]. Changes during the transition of glass into a liquid form the basis of the determination of T_g_ by various techniques, such as differential scanning calorimetry (DSC), thermomechanical analysis (TMA), dynamic mechanical analysis (DMA), rheological methods, dielectric analysis, electron spin resonance (ESR), and nuclear magnetic resonance (NMR), where a combination of various techniques adds value to understanding the nature of glasses and the glass transition [84]. Notably, the structural changes of amorphous materials during the glass transition, in addition to X-ray scattering data, are also confirmed by atomic force microscopy [85,86] and the analysis of fifth-order susceptibility [87]. As Table 1 reveals, the main result of configuron formation in an amorphous material is the shift of atoms from the first coordination shell, regardless of the type of bonding. This means that the analysis of the maxima (PDF_max_) and minima (PDF_min_) of X-ray pair distribution functions is one of the most informative tools that allow for revealing the glass transition and structural changes that accompany this transformation [19,25,88,89,90,91,92,93,94]. Figure 7 demonstrates the stepwise changes in the PDF_min_ based on the data of amorphous Ti_2_Ni taken from [25], along with the changes in the Hausdorff-Besicovitch dimensionality of the set of configurons D, which are of future research interest for amorphous silica. 

A slower cooling of a melt results in a lower T_g_ (this is not shown in Figure 5). Below the melting point T_m_, any liquid tends to crystallize and, with exceptions, such as that of atactic polymers, crystallizes upon sufficiently slow cooling. Crystallization can be avoided kinetically by increasing the cooling rate, although, at any final cooling rate q, the supercooled liquid will contain a certain fraction, denoted here by x, of the crystallized material. The critical cooling rate for glass production is defined as the minimum cooling rate at which the degree of crystallinity of the frozen liquid does not exceed a certain critical value x_c_, where, for good glasses, x_c_ ~ 10^−6^ − 10^−2^ [95]. In an ideal amorphous material, for which x = 0, the percolation threshold is determined using the Scher and Zallen invariant f_c_ = ϑ_c_; however, in a real material, a smaller part of the volume (1 − x) is available for the development of clusters that are made up out of configurons. Similarly, the percolation threshold is similar to the system polydisperse particles [96] and is modified to be f_c_ = ϑ_c_ (1 − x). The fraction of the volume of the crystallized material can be found according to the Kolmogorov–Avrami theory [97,98], where at a constant nucleation rate I_v_, the growth rate of the crystalline phase u and temperature T depends on time t according to x ≅ π I_v_ u^3^ t^4^/3. Thus, the glass transition temperature depends on the logarithm of the cooling rate T_g_= H_c_/{S_c_ + R ln[[1 − f_c_ + π I_v_ (T_m_ − T_g_)^4^ u^3^/3q^4^]/f_c_]} [44,46], which is known experimentally [7]. A proper account of relaxation effects in amorphous materials shows that T_g_ cannot be decreased indefinitely by decreasing the cooling rate and the glass transition temperature interval is quite limited [24]. Kinetically controlled shifts of T_g_ within a relatively narrow temperature interval by the decrease of cooling rate q are thus explained, revealing the logarithmic dependence of T_g_ on q within CPT. The nucleation of bonds also occurs between T_m_ and T_n+_ and not only below T_m_ [36,80]. Consequently, clusters of bound colloids are built below T_n+_, and when the number of new bonds attains the percolation threshold, the crystallization occurs at T_m_ if the liquid is slowly cooled instead of being quenched. 

The above analysis of structural changes of an amorphous material during the glass transition reveals that the transition occurs continuously and that it is similar to second-order phase transformations. The fractal dimensional configuron phase formed above T_g_ is responsible for the liquid-like behavior of the material. Although the transition is continuous and reversible, vitrification occurs differently when cooling the liquid down as no configuron clusters are available to form or disintegrate in the melt. At the same time, the CPT shows a continuous two-exponential function for the viscosity, which provides a rather exact description of viscosity–temperature relationships for both amorphous silica and other materials [24,44,75,99]. However, it should be emphasized that CPT with the equations used above operate with the volume of bonds (V_0_) and configurons (V) and not with the so-called excess or free volume of the material, which is the specific volume of the material (the molecular weight divided by the density) minus the volume of molecules, which is typically assumed to be the volume of hard spheres representing atoms. In line with that, we recall that Doremus (characterizing the predictive ability of viscosity models) accentuated that the bonding between molecules, defects, and the structure are much more important than the free volume [100].

## 5. Conclusions

The role of structural changes during the glass transition is of primary importance to understand the reasons behind the drastic changes of amorphous materials’ behavior due to an increase in temperature when crossing T_g_. The higher the temperature, the more chemical bonds are broken and, thus, the material’s building blocks (either atoms or molecules) become increasingly free to move, gradually changing the solid-like behavior to a more gas-like type behavior. Based on the Kantor–Webman theorem, we conclude that when the percolation threshold for unbound blocks is achieved, then the rigidity threshold of the material is also achieved and, thus, the properties of materials qualitatively change from the solid-like to liquid-like. The crossing of the threshold is continuous; therefore, it is almost an undetectable structural change of the atomic system, i.e., the spatial distribution of building blocks of the material. However, the structural change can be readily revealed for the bonding system instead of the atomic one. The system of configurons, which are broken bonds with an account of relaxation effects near the bond broken, is a point-like system that resembles a gaseous system up to a temperature close to T_g_. The set theory characterizes this system as a set with a Hausdorff dimensionality of 0. Exactly at T_g_, the system of configurons forms a macroscopic percolation cluster for the first time, which is known to be a fractal that is characterized by the Hausdorff dimensionality 2.55 ± 0.05. Thus, the set of configurons changes its dimensionality in a stepwise manner from 0 to 2.55 ± 0.05 at T_g_, which can be interpreted as a kind of symmetry change characterizing the structural difference between a glass and a melt. The qualitative difference between glasses and melts, which could not be revealed for the atomic system, becomes obvious for the system of chemical bonds, which resembles viewing the real image as a positive photograph instead of a negative image on the photographic film.

## Figures and Tables

**Table 1:** Examples of materials with different types of bonding and the corresponding configurons.

Bond Type	Substance	Bond Energy (kJ/mol)	Configuron Description	Microscopic Result of Configuron Formation
Covalent	SiO_2_	443	A Si–O broken bond with neighboring adjustments	A shift by one or more atoms from the first coordination shell
Ionic	CuF_2_	2591	A Cu–F broken bond with neighboring adjustments	A shift by one or more atoms from the first coordination shell
Metallic	Fe	407	A displacement of an atom out of the first coordination shell with neighboring adjustments	A shift by one or more atoms from the first coordination shell
Van der Waals	Ar	7.6	A broken Ar–Ar bond with neighboring adjustments	A shift by one or more atoms from the first coordination shell
Hydrogen	H_2_O	50	A broken hydrogen bond with neighboring adjustments	A shift by one or more atoms from the first coordination shell

## Data Availability

Data supporting reported results are available from the authors.

## References

[1] Mysen B., Richet P. (2019). Silicate Glasses and Melts.

[2] Cheng S. (2021). New interpretation of X-ray diffraction pattern of vitreous silica. Ceramics.

[3] Cheng S. (2021). Viscosity-Temperature relation based on the evolution of medium-range structure of silica. J. Non-Cryst. Solids.

[4] Takada A. (2009). New geometrical modelling of B_2_O_3_ and SiO_2_ glass structures. Phys. Chem. Glasses Eur. J. Glass Sci. Technol. B.

[5] Takada A. (2008). Computer simulation models of glass structure. Phys. Chem. Glasses Eur. J. Glass Sci. Technol. B.

[6] Takada A., Richet P., Catlow C.R.A., Price G.D. (2007). A molecular dynamics simulation of complex structural changes in amorphous silica at high temperatures. Phys. Chem. Glasses Eur. J. Glass Sci. Technol. B.

[7] Zarzycki J. (1982). Glasses and the Vitreous State.

[8] Varshneya A.K. (2006). Fundamentals of Inorganic Glasses.

[9] Dyre J.P. (2006). Colloquium: The glass transition and elastic models of glass-forming liquids. Rev. Mod. Phys..

[10] Angell C.A. (2008). Glass-formers and viscous liquid slowdown since David Turnbull: Enduring puzzles and new twists. MRS Bull..

[11] Klinger M.I. (2013). Glassy Disordered Systems: Glass Formation and Universal Anomalous Low-Energy Properties.

[12] Tournier R.F. (2012). Thermodynamic and kinetic origins of the vitreous transition. Intermetallics.

[13] Tanaka H. (2013). Importance of many-body orientational correlations in the physical description of liquids. Faraday Discuss..

[14] Nemilov S.V. (2018). On the possibility of calculating entropy, free energy, and enthalpy of vitreous substances. Entropy.

[15] Sanditov D.S., Ojovan M.I., Darmaev M.V. (2020). Glass transition criterion and plastic deformation of glass. Phys. B Condens. Matter.

[16] Kittel C. (1996). Introduction to Solid State Physics.

[17] Tournier R.F. (2014). Fragile-to-Fragile liquid transition at Tg and stable-glass phase nucleation rate maximum at the Kauzmann temperature TK. Phys. B Condens. Matter.

[18] Sanditov D.S. (2014). A criterion for the glass-liquid transition. J. Non-Cryst. Solids.

[19] Louzguine-Luzgin D.V., Chen N., Churymov A.Y., Battezzati L., Yavari A.R. (2014). Role of different factors in the glass-forming ability of binary alloys. J. Mater. Sci..

[20] Tournier R.F. (2016). Glass phase and other multiple liquid-to-liquid transitions resulting from two-liquid competition. Chem. Phys. Lett..

[21] Continentino M.A. (2017). Topological phase transitions. Phys. B Condens. Matter.

[22] Zheng Q., Zhang Y., Montazerian M., Gulbiten O., Mauro J.C., Zanotto E.D., Yue Y. (2019). Understanding glass through differential scanning calorimetry. Chem. Rev..

[23] Tournier R.F. (2019). First-order transitions in glasses and melts induced by solid siperclusters nucleated and melted by homogeneous nucleation instead of surface melting. Chem. Phys..

[24] Sanditov D.S., Ojovan M.I. (2019). Relaxation aspects of the liquid–glass transition. Physics-Uspekhi.

[25] Ojovan M.I., Louzguine-Luzgin D.V. (2020). Revealing structural changes at glass transition via radial distribution functions. J. Phys. Chem. B.

[26] Bennett T.D., Tan J.C., Yue Y.Z., Baxter E., Ducati C., Terrill N.J., Yeung H.H.M., Zhou Z., Chen W., Henke S. (2015). Hybrid glasses from strong and fragile metal-organic framework liquids. Nat. Commun..

[27] Medvedev N.N., Geider A., Brostow W. (1990). Distinguishing liquids from amorphous solids: Percolation analysis on the Voronoi network. J. Chem. Phys..

[28] Evteev A.V., Kosilov A.T., Levchenko E.V. (2004). Atomic mechanisms of pure iron vitrification. J. Exp. Theor. Phys..

[29] Ojovan M.I. (2004). Glass formation in amorphous SiO_2_ as a percolation phase transition in a system of network defects. J. Exp. Theor. Phys. Lett..

[30] Fischer H.E., Barnes A.C., Salmon P.S. (2006). Neutron and X-ray diffraction studies of liquids and glasses. Rep. Prog. Phys..

[31] Soper A.K. (2013). The radial distribution functions of water as derived from radiation total scattering experiments: Is there anything we can say for sure?. ISRN Phys. Chem..

[32] Richet P., Conradt R., Takada A., Dyon J. (2021). Encyclopedia of Glass Science, Technology, History, and Culture.

[33] Ojovan M.I. (2021). The Modified random network (MRN) model within the configuron percolation theory (CPT) of glass transition. Ceramics.

[34] Fischer E.W., Meier G., Rabenau T., Patkowski A., Steffen W., Thönnes W. (1991). Density fluctuations around the glass-transition of low molecular weight glass-forming liquids. J. Non-Cryst. Solids.

[35] Bakai A.S., Fischer E.W. (2004). Nature of long-range correlations of density fluctuations in glass-forming liquids. J. Chem. Phys..

[36] Yue Y. (2004). Experimental evidence for the existence of an ordered structure in a silicate liquid above its liquidus temperature. J. Non-Cryst. Sol..

[37] Ozhovan M.I. (1993). Dynamic uniform fractals in emulsions. J. Exp. Theor. Phys..

[38] Angell C.A. (1968). Oxide glasses in light of the ’Ideal glass’ concept. I. General aspects: Ideal and non-ideal transitions. J. Am. Ceram. Soc..

[39] Angell C.A., Richards B.E., Velikov V. (1999). Simple glass-forming liquids: Their definition, fragilities, and landscape excitation profiles. J. Phys. B Condens. Matter.

[40] Angell C.A., Wong J.J. (1970). Structure and glass transition thermodynamics of liquid zinc chloride from far-infrared, Raman, and probe ion electronic and vibrational spectra. Chem. Phys..

[41] Angell C.A., Rao K.J. (1972). Configurational excitations in condensed, and the “bond lattice” model for the liquid-glass transition. J. Chem. Phys..

[42] Egami T., Miller M., Liaw P. (2008). Atomistic theory of metallic liquids and glasses. Bulk Metallic Glasses.

[43] Kantor Y., Webman I. (1984). Elastic properties of random percolating systems. Phys. Rev. Lett..

[44] Ozhovan M.I. (2006). Topological characteristics of bonds in SiO_2_ and GeO_2_ oxide systems upon a glass-liquid transition. J. Exp. Theor. Phys..

[45] Ojovan M.I., Lee W.E. (2010). Connectivity and glass transition in disordered oxide systems. J. Non-Cryst. Solids.

[46] Ashkroft N.W., Mermin N.D. (1976). Solid State Physics.

[47] Tanaka H. (2005). Two-order-parameter model of the liquid-glass transition. I. Relation between glass transition and crystallization. J. Non-Cryst. Solids.

[48] Sun G.Q. (2016). Pattern transitions in spatial epidemics: Mechanisms and emergent properties. Phys. Life Rev..

[49] Li L. (2015). Patch invasion in a spatial epidemic model. Appl. Math. Comput..

[50] Dumonteil E. (2013). Spatial extent of an outbreak in animal epidemics. Proc. Natl. Acad. Sci. USA.

[51] Houchmandzadeh B. (2008). Neutral clustering in a simple experimental ecological community. Phys. Rev. Lett..

[52] Houchmandzadeh B. (2002). Clustering of diffusing organisms. Phys. Rev. E.

[53] Young W.R. (2001). Reproductive pair correlations and the clustering of organisms. Nature.

[54] Cox J.T., Griffeath D. (1985). Occupation times for critical branching Brownian motions. Ann. Probabil..

[55] Taraskin S.N., Perez-Reche F.J. (2013). Effects of variable-state neighborhoods for spreading synergystic processes on lattices. Phys. Rev. E.

[56] Dumonteil E., Bahran R., Cutler T., Dechenaux B., Grove T., Hutchinson J., McKenzie G., McSpaden A., Monange W., Nelson M. (2021). Patchy nuclear chain reactions. Commun. Phys..

[57] Isichenko M.B. (1992). Percolation, statistical topography, and transport in random media. Rev. Mod. Phys..

[58] Frenkel J. (1946). Kinetic Theory of Liquids.

[59] Bolmatov D., Brazhkin V.V., Trachenko K. (2012). The phonon theory of liquid thermodynamics. Sci. Rep..

[60] Brazhkin V., Trachenko K. (2012). What separates a liquid from a gas?. Physics Today.

[61] Trachenko K., Brazhkin V.V. (2016). Collective modes and thermodynamics of the liquid state. Rep. Prog. Phys..

[62] Trachenko K. (2017). Lagrangian formulation and symmetrical description of liquid dynamics. Phys. Rev. E.

[63] Benigni P. (2021). CALPHAD modeling of the glass transition for a pure substance, coupling thermodynamics and relaxation kinetics. Calphad.

[64] Scher H., Zallen R. (1970). Critical density in percolation processes. J. Chem. Phys..

[65] Mazurin O.V. (2007). Problems of compatibility of the values of glass transition temperatures published in the world literature. Glass Phys. Chem..

[66] Tournier R.F., Ojovan M.I. (2021). Undercooled phase behind the glass phase with superheated medium-range order above glass transition temperature. Phys. B Condens. Matter.

[67] Ojovan M.I. (2008). Configurons: Thermodynamic parameters and symmetry changes at glass transition. Entropy.

[68] Hunt A. (1992). A Purely kinetic justification for application of Ehrenfest theorems to the glass transition. Solid State Commun..

[69] Tournier R.F., Ojovan M.I. (2021). Dewetting temperatures of prefrozen and grafted layers in ultrathin films viewed as melt-memory effects. Phys. B Condens. Matter.

[70] Rincon J.M. Structure and microstructure of glasses and geopolymers under electron microscopies. Proceedings of the 2nd Workshop-Symposium Vitro Geowastes: Vitrification, Geopolymerization, Wastes Management and Circular Economy.

[71] Rincón J.M. (2016). Vitreous and ceramic processing for the recycling of industrial wastes. Key Eng. Mater..

[72] Rincón J.M., Casasola R. (2015). TEM Replica of fluor-miserite glassceramic. Mater. Technol..

[73] Rincon J., Duran A. (1982). Separacion de Fasesen Vidrios.

[74] Mazurin O.V., Porai-Koshits E.A. (1984). Phase Separation in Glass.

[75] Ojovan M.I. (2013). Ordering and structural changes at the glass–liquid transition. J. Non-Cryst. Solids.

[76] Angel C.A. (2002). Calorimetric studies of the energies landscapes of glassformers by hyper-quenching methods. J. Therm. Anal. Calorim..

[77] Jaeger G. (1998). The Ehrenfest classification of phase transitions: Introduction and evolution. Arch. Hist. Exact Sci..

[78] Patashinskioe A.Z., Pokrovskioe V.L. (1979). Fluctuation Theory of Phase Transitions.

[79] Landau L.D., Lifshitz E.M. (2013). Statistical Physics.

[80] Tournier R.F., Ojovan M.I. (2021). Building and breaking bonds by homogenous nucleation in glass-forming melts leading to transitions in three liquid states. Materials.

[81] Holmlid L. (2007). Direct observation of circular Rydberg electrons in a Rydberg matter surface layer by electronic circular dichroism. J. Phys. Condens. Matter..

[82] Aasen T.H., Zeiner-Gundersen D.H., Zeiner-Gundersen S., Ohlckers P., Wanget K. (2021). A condensed excited (Rydberg) matter: Perspective and applications. J. Clust. Sci..

[83] Cunningham D.W. (2016). Set Theory: A First Course.

[84] Witek M., Krzystyniak M., Romanelli G., Witczak T. (2021). Glass transition in rice pasta as observed by combined neutron scattering and time-domain NMR. Polymers.

[85] Stanzione J.F., Strawhecker K.E., Wool R.P. (2011). Observing the twinkling fractal nature of the glass transition. J. Non-Cryst. Solids.

[86] Wool R.P. (2008). Twinkling fractal theory of the glass transition. J. Polym. Sci. B Polym. Phys..

[87] Albert S., Bauer T., Michl M., Biroli G., Bouchaud J.-P., Loidl A., Luckenheimer P., Tourbot R., Wiertel-Gasquet C., Ladieu F. (2016). Fifth-order susceptibility unveils growth of thermodynamic amorphous order in glass-formers. Science.

[88] Louzguine-Luzgin D.V., Seki I., Ketov S.V., Louzguina-Luzgina L.V., Polkin V.I., Chen N., Fecht H., Vasiliev A.N., Kawaji H. (2015). Glass-transition process in an Au-based metallic glass. J. Non-Cryst. Solids.

[89] Louzguine-Luzgin D.V., Georgarakis K., Tsarkov A., Solonin A., Honkimaki V., Hennet L., Yavari A.R. (2015). Structural changes in liquid Fe and Fe-B alloy on cooling. J. Mol. Liq..

[90] Wang Z., Chen C.L., Ketov S.V., Akagi K., Tsarkov A.A., Ikuhara Y., Louzguine-Luzgin D.V. (2018). Local chemical ordering within the incubation period as a trigger for nanocrystallization of a highly supercooled Ti-based liquid. Mater. Des..

[91] Louzguine-Luzgin D.V., Belosludov R., Yavari A.R., Georgarakis K., Vaughan G., Kawazoe Y., Egami T., Inoue A. (2011). Structural basis for supercooled liquid fragility established by synchrotron-radiation method and computer simulation. J. Appl. Phys..

[92] Louzguine-Luzgin D.V., Miyama M., Nishio K., Tsarkov A.A., Greer A.L. (2019). Vitrification and nanocrystallization of pure liquid Ni studied using molecular-dynamics simulation. J. Chem. Phys..

[93] Louzguine-Luzgin D.V. (2014). Vitrification and devitrification processes in metallic glasses. J. Alloys Compd..

[94] Georgarakis K., Louzguine-Luzgin D.V., Antonowicz J., Vaughan G., Yavari A.R., Egami T., Inoue A. (2011). Variations in atomic structural features of a supercooled Pd-Ni-Cu-P glass forming liquid during in situ vitrification. Acta Mater..

[95] Uhlmann D.R. (1972). A kinetic treatment of glass formation. J. Non-Cryst. Solids.

[96] Ozhovan M.I., Semenov K.N. (1992). Percolation in a system of polydispersed particles. J. Exp. Theor. Phys..

[97] Uhlmann D.R., Hays J.F., Turnbull D. (1966). The effect of high pressure on crystallization kinetics with special reference to fused silica. Phys. Chem. Glasses.

[98] Fanfoni M., Tomellini M. (1998). The Johnson-Mehl-Avrami-Kolmogorov model: A brief review. Il Nuovo Cimento D.

[99] Ojovan M. (2020). On viscous flow in glass-forming organic liquids. Molecules.

[100] Doremus R.H. (2002). Viscosity of silica. J. Appl. Phys..

